# A Biorefinery Approach Integrating Lipid and EPS Augmentation Along with Cr (III) Mitigation by *Chlorella minutissima*

**DOI:** 10.3390/cells13242047

**Published:** 2024-12-11

**Authors:** Sonia Choudhary, Mansi Tiwari, Krishna Mohan Poluri

**Affiliations:** 1 Department of Biosciences and Bioengineering, Indian Institute of Technology Roorkee, Roorkee 247667, India; schoudhary1@bt.iitr.ac.in (S.C.); mansi_t@bt.iitr.ac.in (M.T.); 2 Centre for Transportation System, Indian Institute of Technology Roorkee, Roorkee 247667, India

**Keywords:** *Chlorella minutissima*, chromium (Cr (III)), bioremediation, FAME, lipidomics, oxidative stress, EPS, green fuel

## Abstract

The quest for cleaner and sustainable energy sources is crucial, considering the current scenario of a steep rise in energy consumption and the fuel crisis, exacerbated by diminishing fossil fuel reserves and rising pollutants. In particular, the bioaccumulation of hazardous substances like trivalent chromium has not only disrupted the fragile equilibrium of the ecological system but also poses significant health hazards to humans. Microalgae emerged as a promising solution for achieving sustainability due to their ability to remediate contaminants and produce greener alternatives such as biofuels. This integrated approach provides an ambitious strategy to address global concerns pertaining to economic stability, environmental degradation, and the energy crisis. This study investigates the intricate defense mechanisms deployed by freshwater microalgae *Chlorella minutissima* in response to Cr (III) toxicity. The microalga achieved an impressive 92% removal efficiency with an IC_50_ value of 200 ppm, illustrating its extraordinary resilience towards chromium-induced stress. Furthermore, this research embarked on thorough explorations encompassing morphological, pigment-centric, and biochemical analyses, aimed at revealing the adaptive strategies associated with Cr (III) resilience, as well as the dynamics of carbon pool flow that contribute to enhanced lipid and extracellular polysaccharide (EPS) synthesis. The FAME profile of the biodiesel produced complies with the benchmark established by American and European fuel regulations, emphasizing its suitability as a high-quality vehicular fuel. Elevated levels of ROS, TBARS, and osmolytes (such as glycine-betaine), along with the increased activity of antioxidant enzymes (CAT, GR, and SOD), reveal the activation of robust defense mechanisms against oxidative stress caused by Cr (III). The finding of this investigation presents an effective framework for an algal-based biorefinery approach, integrating pollutant detoxification with the generation of vehicular-quality biodiesel and additional value-added compounds vital for achieving sustainability under the concept of a circular economy.

## 1. Introduction

In the preceding decade, a significant surge in energy consumption, coupled with a fuel crisis intensified by the depletion of fossil fuel reserves and exacerbated by the significant rise of global warming, presents an unavoidable global challenge [1,2]. Certainly, in light of the trivial surge in global demographics, it is projected that energy demands will soar by 47% by 2050, accompanied by a concerning rise in atmospheric carbon emissions and the accumulation of pollutants in subterranean water sources as a result of rapid industrial development [3,4]. This endeavor demands the embrace of sustainable, carbon-conscious practices to foster social and economic flourishing within the community [5]. A promising solution lies in harnessing bioagents like microalgae for the management of waste and the reclamation of resources, fostering a circular methodology for producing renewable energy via biorefinery methods [6]. These holistic strategies confront the worldwide crisis while concurrently bolstering environmental security, thereby fortifying the pillars of a circular economy [7]. Microalgae are able to exploit wastewater as an economically viable nutrient reservoir, yielding biomass that may serve as a precursor for biofuel production [8,9,10]. Nevertheless, although wastewaters can facilitate algal proliferation, the presence of toxic contaminants, including heavy metals, constrains their applicability for most of algal strains [11,12].

Over the past few years, a wide range of hazardous pollutants and chemicals have emerged as a significant global threat. These contaminants include various compounds such as polycyclic aromatic hydrocarbons (PAHs), petroleum hydrocarbons, chlorinated solvents, pharmaceutical residues, agricultural chemicals, and more [13,14,15,16]. Among these, the reckless release of toxic heavy metals has not only disrupted the intricate equilibrium of ecological systems but also poses considerable health hazards to humans. Chromium stands out as a particularly formidable pollutant that is widely used across industries, including chemical manufacturing, automotive, metallurgy, and even in the chic landscape of fashion and textiles [17,18]. It exists in various oxidation states, including divalent, trivalent, pentavalent, and hexavalent, each with differing levels of toxicity [19]. Yet, within the realm of nature, it is solely trivalent and hexavalent chromium that exhibit stability. Trivalent chromium, Cr (III), is commonly found in wastewater from industries including leather tanning, dye production, wood preservation, and electroplating [20,21]. Although Cr (III) is an essential trace element vital for the breakdown of sugars and lipids, prolonged exposure can lead to cancer, genetic disorder/mutations, and developmental anomalies in humans, as well as detrimental impacts on both aquatic and terrestrial life forms [22,23]. Chronic exposure to Cr (III) can cause skin ailments, breathing difficulties, and neurological disorders, especially via skin contact and inhalation [24], while long-term studies indicate that chromium compounds may also disrupt reproductive health and cause systemic diseases [25].

As most heavy metals exhibit hydrophilic properties, their movement through the partially lipophilic biological membrane encasing the cell is facilitated by particular proteins. These metals can infiltrate the cell through receptor-mediated endocytosis after associating with low molecular weight thiols such as glutathione, cysteine, and homocysteine [26,27,28,29]. These thiols interact with heavy metals via their sulfhydryl (-SH) groups, thereby promoting their transportation and detoxification within the cellular environment. Similarly, Cr (III) readily binds to carboxyl, hydroxyl, amino, and phosphate groups present on the microalgal cellular surface, which play a crucial role in biosorption [30,31]. Despite the pronounced toxicity of Cr (III), certain microalgal species exhibit resilience and adaptability to toxic conditions, attributed to their metabolic versatility [32,33]. A common adaptive response in algae under such abiotic stress involves redirecting carbon resources to produce energy dense compounds such as carbohydrates and lipids [34,35]. This strategy not only reduces oxidative burden in algal cells but also serves as a resource for the ecological development of microalgae derived biofuels. Utilizing microalgae for wastewater purification presents an eco-friendly and budget-friendly solution. This approach allows the simultaneous removal of the concurrent retrieval of trace metals and the cultivation of microalgal biomass tailored for green fuel generation and multifaceted industrial application. Besides biofuels, the stress-driven generation of additional valuable substances, including extracellular polysaccharides (EPS), offers a captivating biorefining framework for biorefining the biodiesel production [36]. Contemporary research has yielded biochemical substantiation regarding the remediation of Cr (III) via microalgae and the production of biodiesel; however, there remains a paucity of investigation into the generation of EPS [37].

This study aims to develop a holistic strategy for the phycoremediation of Cr (III) while concurrently boosting lipid generation for biodiesel production utilizing *Chlorella minutissima*, emphasizing the physiological and biochemical changes. An extensive temporal examination lasting 12 days was performed to gauge the alterations in the biochemical makeup of the microalga, highlighting its adaptive mechanism for tolerating Cr (III) toxicity. The study probes into Cr (III) induced alterations in lipid compositions, EPS levels, and the associated antioxidant response mechanisms to reveal the principal stress adaptation techniques utilized by *C. minutissima*. Furthermore, the heightened EPS fraction under Cr (III) stress implies an integrated regulatory system that harmonizes lipid and polysaccharide production to alleviate Cr (III) toxicity. In summary, the investigation underscores the bioprospecting potential of an inherently microalgae-based biorefinery approach, integrating green fuel creation, production of EPS, and the eco-friendly remediation of hazardous chromium.

## 2. Materials and Methods

### 2.1. Cultivation and Maintenance of Microalgal Cultures

*Chlorella minutissima* (MCC-27) was obtained from the preservation facility at the Indian Agricultural Research Institute located in New Delhi. The strain underwent an 8 day cultivation process in Bold’s Basal Medium (BBM) within a pH range of 7.2 to 7.4. This growth phase was conducted under continuous light (200 μmol m^−2^ s^−1^) at a temperature maintained at 26 ± 2 °C while the culture was stirred at a speed of 180 rpm. The formulation of BBM is detailed in Tables S1 and S2 [34].

### 2.2. Toxicity Assessment of Cr (III)

In order to ascertain the toxicological effects of Cr (III) on *C. minutissima*, the IC_50_ value (inhibitory concentration) was established by cultivating microalgae in a range of Cr (III) concentrations. A spirited amalgamation of chromium nitrate (Cr(NO_3_)) at 10 g/L in Milli-Q water was utilized with Cr (III) levels escalating from 0 to 300 ppm, as cells were introduced at their growth peak with an initial optical density of 0.3, thriving for 96 h [34]. Cell inhibition was estimated by employing the following formula:

$$Cell\;Inhibition\;(\%)=100-\left[\left(\frac{OD\;of\;Cr\;\left(III\right)treated\;cells}{OD\;of\;control\;cells}\right)\right]\times 100$$

### 2.3. Cellular Growth and Biomass Estimation

Leveraging the determined IC_50_ value, this investigation assessed the growth of *C. minutissima* under the effect of 100 and 200 ppm Cr(NO_3_) incorporated into BBM for a duration of 12 days. A control cohort, which did not experience Cr (III) exposure, was retained for comparative analysis. The assessment of cellular growth was performed by evaluating OD at 750 nm at 24 h intervals [34]. The harvested biomass was subjected to lyophilization and was subsequently evaluated for dry cell weight (DCW) at intervals of 72 h.

### 2.4. Analysis of Residual, Adsorbed, and Accumulated Cr (III) Ions

The astonishing prowess of *C. minutissima* to proficiently eradicate Cr (III) was examined by quantifying the residual Cr (III) in the medium at 72 h intervals through the use of microwave plasma atomic emission spectrometry (Agilent 4210-MP-AES, Santa Clara, CA, USA). Cr (III) removal (%) was determined by using the equation mentioned below:

$$Cr\;(III)\;removal\left(\%\right)=\frac{\left(C_{i}-C_{f}\right)}{C_{i}}\times 100$$

C_i_ denotes the initial concentration of Cr (III), whereas C_f_ signifies the leftover Cr (III) concentration in the media post-treatment.

To ascertain the quantity of Cr (III) adsorbed onto the cellular matrices, experimental samples were formulated by incubating 100 mg of fresh biomass alongside 10 mM ethylenediamine tetraacetic acid (EDTA) for a duration of 20 min (Tripathi et al. 2021, [29]). Further, samples were subjected to centrifugation, and resultant supernatant was subsequently filtered and preserved for examination through MP-AES. After EDTA treatment, the pellet was incubated in concentrated HNO_3_ and 30% H_2_O_2_ for 1 h at 100 °C. After this incubation, the digestate was suitably diluted and filtered for assessing the chromium uptake by microalgal cells [38]. The bio-concentration factor (BCF) for chromium (III) within the delicate embrace of *C. minutissima* was calculated employing the following formula:

$$BCF=\frac{Total\;Cr\;\left(III\right)\;adsorbed\;and\;accumulated\;by\;biomass}{Initial\;Cr\;\left(III\right)concentration\;in\;the\;media}$$

### 2.5. Photosynthetic Pigment Estimation

Pigments were extracted by immersing an equivalent volume of fresh culture in pure methanol at a temperature of 45 °C while perpetually agitating at 180 rpm for a time span of 24 h in the absence of light. Post-incubation, the pigment concentrations in the resultant supernatant were analyzed by recording absorbance at 665, 652, and 470 nm. The pigment concentrations (μg/mL) were derived employing the following equations [34,39]:

$$Chlorophyll\;a\;(µg/mL)=16.71\times A\;\left(665\;nm\right)-9.16\times A\;(652\;nm)$$

$$Chlorophyll\;b\;(µg/mL)=34.09\times A(652\;nm)-15.28\times A\;\left(665\;nm\right)$$

$$Carotenoid\;(µg/mL)=\left[\left(1000\times A\;(470\;nm\right))-\left(1.63\times Chl\;a\right)-\left(104.9\times Chl\;b\right)\right]/221$$

Total Chlorophyll (µg/mL)=Chlorophyll a+Chlorophyll b

$$PS\;II\;efficiency=\frac{Carotenoid}{Total\;chlorophyll}$$

### 2.6. Biochemical Analysis

The biochemical variations between the control and Cr (III) treated biomass were scrutinized through the evaluation of total protein, carbohydrate, and lipid ratios. A 2:1 *w*/*v* blend of chloroform and methanol was utilized to extract total lipids, while the cells stained with Nile red were examined through fluorescence microscopy to evaluate the presence of lipid droplets [40,41]. The total protein content was gauged using the Bradford method, leveraging bovine serum albumin (BSA) (Sico Research Laboratories (SRL), Mumbai, India) as a standard, while the phenol–sulphuric method was used to determine carbohydrate levels in the freeze-dried biomass [37,41,42].

### 2.7. Lipid Content Profiling by ^1^H-NMR Spectroscopy

For ^1^H-NMR analysis, cellular lipids were extracted and purified utilizing the Bligh and Dyer (1959) methodology, followed by dissolution in deuterated chloroform (CDCl_3_) augmented with a 0.03% tetramethylsilane (TMS) standard. The ^1^H-NMR spectra were captured at 298 K using a sophisticated 500 MHz NMR spectrometer (Bruker, Fällanden, Switzerland) implementing the zg30 pulse sequence across 128 acquisitions, encompassing 64 k data points and a deliberate 4 s relaxation interval. Peak assignment and identification were diligently executed using Bruker Topspin 3.5 software [35,43].

### 2.8. Fatty Acid Methyl Ester (FAME) Profiling and Biodiesel Properties

The lipid acquired was subjected to a transesterification reaction using methanolic H_2_SO_4_ at 90 °C for a precise 45 min [10]. Being a strong catalyst, H_2_SO_4_ ensures an efficient conversion of lipids to fatty acid methyl esters [44]. Subsequent to the conclusion of the reaction, the mixtures were allowed to attain room temperature before being mixed with hexane and distilled water in a 1:2 ratio. The lower phase, abundant in FAME, was meticulously extracted in hexane for examination through Gas Chromatography–Mass Spectrometry (GC-MS), featuring a DB5 capillary column with helium as the carrier gas. A sample volume of 1 μL was introduced at 250 °C, with a flow rate of 1 mL/min, initiating at 50 °C and progressively ascending to 180 °C at a pace of 25 °C/min for 1 min, followed by an elevation to 220 °C at 10 °C/min and ultimately achieving 250 °C at 15 °C/min, where it remained for an additional 15 min [45]. The ion source temperature was set at 230 °C to optimize the detection of fatty acids. Furthermore, the biodiesel attributes of the FAME extracted from the lipid were meticulously assessed through theoretical equations, as outlined in Table S3.

### 2.9. Morphology and Surface Characteristics

The effects of Cr (III) exposure on microalgal cellular architecture were scrutinized using a light microscope in conjunction with a field emission scanning electron microscope (FE-SEM) paired with energy-dispersive X-ray (EDX) spectroscopy [34]. Algal suspensions derived from both the control culture and those exposed to Cr (III) were mounted onto glass slides using a 2.5% glutaraldehyde solution. Subsequently, samples were dehydrated using an ethanol gradient from 10% to 100% concentration, followed by gold sputter coating before analysis via FE-SEM. The variation in cell dimensions was assessed employing ImageJ v/1.52 software. Additionally, shifts in surface charge were tracked by measuring the zeta potential (ZP) for both control and experimental samples. To unveil changes in the functional groups on the microalgal cellular surface, freeze-dried biomass was analyzed using Fourier transform infrared (FTIR) spectroscopy across the mid-infrared spectrum (400–4000 cm^−1^) [10]. To assess the chemical states of Cr (III) in the microalgal biomass, X-ray photoelectron (XPS) spectroscopy (PHI 5000 VersaProbe III) (ULVAC-PHI, Inc., Hagisono, Chigasaki, Kanagawa, Japan), was utilized, using the C 1s peak at 284.8 eV as a benchmark for reference [34].

### 2.10. Analysis of Stress-Elicited Metabolites and Antioxidant Enzymes

The detrimental repercussions of Cr (III) biosorption on microalgae were explored through the evaluation of the overall cellular reactive oxygen species (ROS) concentration. This assessment entailed the submersion of the microalgal cells in a fluorescent solution of 2′,7′-dichlorodihydrofluorescein diacetate (H2DCFDA) in dark for 45 min. Following this incubation period, the fluorescence emission and excitation were recorded at 520 and 486 nm, respectively [34].

The thorough quantification of thiobarbituric acid reactive substances (TBARSs) within cellular structures served as a benchmark for assessing lipid peroxidation. A 50 mg fresh biomass was homogenized in 0.1% trichloroacetic acid (TCA), followed by concoction with thiobarbituric acid (TBA) [10]. TBARS content was assessed as follows:

TBARS content=(A (532 nm)−A (600 nm))×EC

where EC (extinction coefficient) value is 1.56 × 10^5^ M/cm for the MDA-TBA abduct.

The proline levels in control and Cr (III) exposed microalgal cells were assessed using an L-proline [0 to 50 µM] calibration curve, with fresh biomass homogenized in 3% sulfosalicylic acid, followed by toluene extraction and precise absorbance measurement at 520 nm [46]. To evaluate the concentration of glycine betaine (GB), a mixture of an acidic solution combined with equal proportions of deionized water and 2 N H_2_SO_4_ was utilized to homogenize the biomass. This was followed by incubation with the chilled K2-I2 reagent and 2-dichlorethane, where the absorbance at 365 nm was analyzed using a 0–20 µM reference curve for GB concentrations [47]. 

The enzyme extract intended for the quantification of antioxidant activity was obtained through the homogenization of fresh biomass in a lysate buffer. The catalytic performance of catalase (CAT) was analyzed by quantifying the H_2_O_2_ consumption with absorbance changes observed at 240 nm for a time period of 3 min [35]. The activity of ascorbate peroxidase (APX) was assessed by observing the gradual alteration in absorbance at 290 nm while measuring the degradation of ascorbic acid in the presence of H_2_O_2_ [34]. In a similar manner, the role of glutathione reductase (GR) was investigated by tracking the conversion of NADPH to NADP at 340 nm in relation to glutathione disulfide (GSSG) [48]. The analysis of superoxide dismutase (SOD) activity included the photoreduction of nitroblue tetrazolium (NBT) through the utilization of riboflavin and methionine [10].

### 2.11. Estimation of Carbonic Anhydrase Activity

The significance of carbonic anhydrase in the realm of microalgal cells was unveiled by probing into esterase activity. To summarize the approach, 0.5 g of microalgal biomass was merged with 1 mL of 10 mM Tris-HCl buffer (pH 7.4) to concoct the cellular extract. A 200 µL aliquot of this extract was then blended with 1.8 mL of Tris-HCl buffer and 1 mL of 3 mM p-nitrophenyl acetate. The absorbance was assessed at 400 nm and observed over a span of 5 min to exemplify the escalating absorbance consequent to the discharge of p-nitrophenol, which has a molar extinction coefficient (ε) of 15,000 M^−1^ cm^−1^ [35,49].

### 2.12. Extraction and Qualitative/Quantitative Estimation of EPS

The quantification of soluble extracellular polymeric substances (EPS) in the supernatant of both control and Cr (III) treated algal cultures was executed by reducing the supernatant to a quarter of its original volume at 65 °C using a vacuum concentrator, followed by ethanol precipitation to isolate EPS. The samples were then spun in a centrifuge at 12,000 rpm for 40 min at 4 °C [35]. The resulting samples underwent a series of processes including centrifugation, rinsing, freeze-drying, and gravimetric measurement, followed by characterization through infrared spectroscopy and the phenol–sulphuric method for determining total carbohydrate content [35].

### 2.13. Statistical Analysis

The dataset was meticulously analyzed using triplicate samples (n = 3) from three distinct cultures, with results expressed as means and standard deviations to highlight central trends and variability; credibility was ensured through one-way ANOVA followed by Tukey’s test, denoting significance levels with *p*-values < 0.05, 0.01, and 0.001, represented by *, **, and ***.

## 3. Results

### 3.1. Growth Modulation of C. minutissima Under Cr (III) Stress

**Figure 1:**
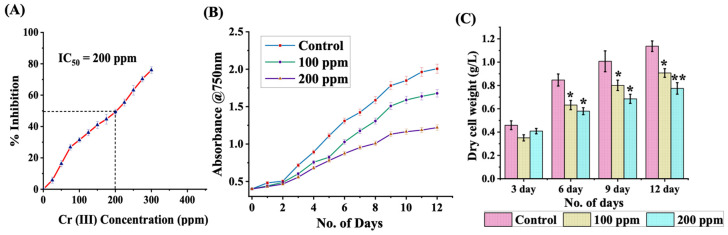
(**A**) The IC_50_ concentration of Cr (III) for *C. minutissima* after 96 h of exposure. (**B**) Growth curve comparison for *C. minutissima* under standard conditions and in the presence of Cr (III) at concentrations of 100 ppm and 200 ppm. (**C**) Changes in dry cell weight of *C. minutissima* over 12 days, with measurements taken every 72 h. The data denotes the mean ± S.D. from three independent replicates, with *p*-values < 0.05 and 0.01 indicated by * and **, respectively.

Bioprospecting algal strains with the proficiency to thrive and endure Cr (III) from contaminated aquatic environments present a sustainable and effective technological avenue, which may also offer supplementary advantages associated with biomass production for bioenergy generation. In order to gauge the fortitude of *C. minutissima* in media augmented with Cr (III), the half-maximal inhibitory concentration (IC_50_) was ascertained by cultivating them across a concentration range of 0–300 ppm for a duration of 96 h (Figure 1A). A comprehensive evaluation of the IC_50_ value of *C. minutissima* in comparison to prior studies suggests that this algal variant exhibits notable resistance to Cr (III) exposure, emphasizing it as a viable candidate in bioremediation efforts (Table S4). Subsequent to the incubation phase, it was recorded that cellular growth exhibited an IC_50_ of 200 ppm for Cr (III). 

Leveraging the ascertained IC_50_ value, *C. minutissima* was cultivated in BBM enriched with 100 and 200 ppm Cr (III) concentration for a span of 12 days, with the intent of scrutinizing the growth patterns of the cultures (Figure 1B). The findings clearly demonstrate that all cultures transitioned into the logarithmic growth phase by day 3, although growth was notably reduced in cultures influenced by Cr (III). Cultures devoid of Cr (III) demonstrated a doubling time that hovered around day 2 to day 3, contrasting with the 100 and 200 ppm Cr (III) treated cultures, which showed a doubling time extending from day 3 to day 4. The accelerated growth phase was sustained until day 10 in both control cells and those exposed to 100 ppm Cr (III). However, in cultures treated with 200 ppm Cr (III), this phase concluded by day 9, culminating in the initiation of the stationary phase on day 10, which was marked by negligible growth. These outcomes reveal that Cr (III) inflicts toxic repercussions on *C. minutissima*, restricting the growth of the microalga. Consequently, reduction in biomass production was documented, which was directly related to the concentration of Cr (III) (Figure 1C). In a comparative analysis, cells treated with 200 ppm Cr (III) demonstrated a notable decrease of around 32% in biomass after 12 days, while cultures with 100 ppm Cr (III) faced ~20% biomass reduction. The control cultures exhibited the zenith of biomass production at 1.13 ± 0.04 g/L, followed closely by those cultivated in 100 ppm Cr (III) at 0.90 ± 0.03 g/L and the 200 ppm Cr (III) cohort at 0.77 ± 0.04 g/L after 12 days of incubation. The observed decrease in biomass was also reflected in the hues of the cultures exposed to Cr (III) (Figure S1).

### 3.2. Cr (III) Removal Dynamics in C. minutissima

**Figure 2:**
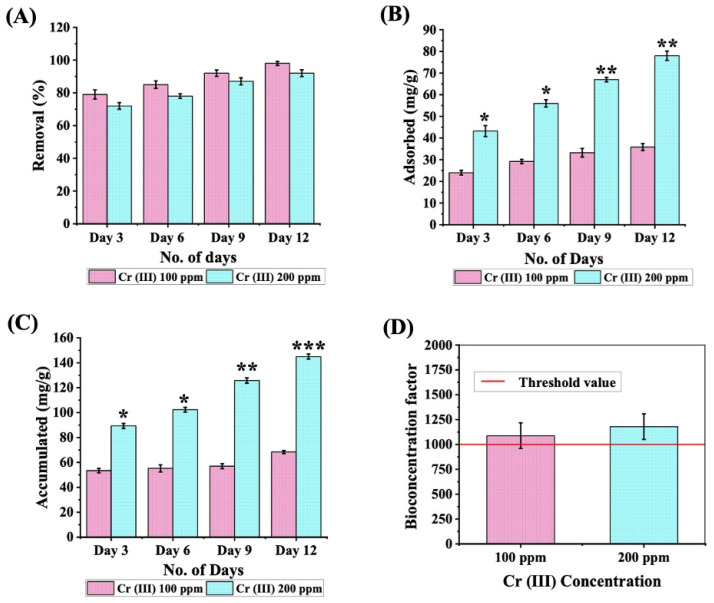
(**A**) Removal efficiency of Cr (III) from the medium over time. (**B**) Time-course analysis of Cr (III) adsorbed on the cell surface of *C. minutissima*. (**C**) Quantification of Cr (III) accumulated within *C. minutissima* cells over 12 days, with measurements taken every 72 h. (**D**) Assessment of the bioconcentration factor for *C. minutissima* after 12 days of exposure to Cr (III) at concentrations of 100 ppm and 200 ppm. The data denotes the mean ± S.D. from three independent replicates, with *p*-values < 0.05, 0.01 and 0.001 represented by *, **, and ***.

The microalgal cells exhibited remarkable resilience to Cr (III) toxicity, steadfastly persisting in their growth, which necessitates a deeper exploration of their removal potential. The dynamic removal trends of Cr (III) at concentrations of 100 and 200 ppm are depicted in Figure 2A. An impressive 70–80% removal was documented in the initial 3 days for both concentrations, accompanied by a continuous improvement in removal efficiency over the 12 day incubation period. By the end of the 12 day incubation stretch, nearly 92% (~184 ppm) of Cr (III) was triumphantly eradicated at the heightened concentration of 200 ppm, while at the modest initial concentration of 100 ppm of Cr (III), the cells adeptly cleared about 98% (~98 ppm) of Cr (III). Further, to gain a profound insight into the myriad of influences on the remediation of Cr (III), it is essential to evaluate the amounts that are adsorbed and absorbed. A chronological exploration of the extracellularly retained Cr (III) on the microalgal cell surface, together with the Cr (III) ions stored within the cells, were analyzed (Figure 2B,C). In this initial period, the rapid removal of Cr (III) is facilitated by passive surface adsorption via an array of functional groups (carboxyl, hydroxyl, amino, phosphate) present on the microalgal surface [30,50]. These functional groups can swiftly engage with Cr (III) ions through electrostatic interactions, ion exchange, and complexation. This initial adsorption phase occurred independently of metabolic activity and accounted for the majority of Cr (III) removal, relying on surface interaction [12]. Following adsorption, the process of active cellular uptake (bioaccumulation) commences, wherein Cr (III) may penetrate cells through mechanisms such as passive diffusion and active transport [51,52]. The findings underscored that till day 3, the cells exhibited remarkable adsorption levels (43.24 ± 2.53 mg/g) of Cr (III) ions. The cells swiftly ensnared the captured ions, leading to a significant surge of diminished Cr (III) ions within the microalgal cells. As the 12-day incubation period concluded, the peak adsorbed Cr (III) content was noted at 35.84 ± 1.57 mg/g and 78 ± 2.12 mg/g for concentrations of 100 and 200 ppm Cr (III), respectively. A similar pattern emerged in the accumulation dynamics, where 68.4 ± 1.23 mg/g and 145 ± 2.07 mg/g of Cr (III) for 100 and 200 ppm concentrations was observed within the cells. The results clearly illustrate that both bioadsorption and bioaccumulation significantly contributed to the attenuation of reasonable amounts of Cr (III) from the cultures. Additionally, the significant uptake and retention of Cr (III) by *C. minutissima* was confirmed through an analysis of the bioconcentration factor (BCF). At Cr (III) concentrations of 100 and 200 ppm, the BCF values surpassed 1000, establishing *C. minutissima* as a highly efficient hyperaccumulator of trivalent chromium (Figure 2D).

**Figure 3:**
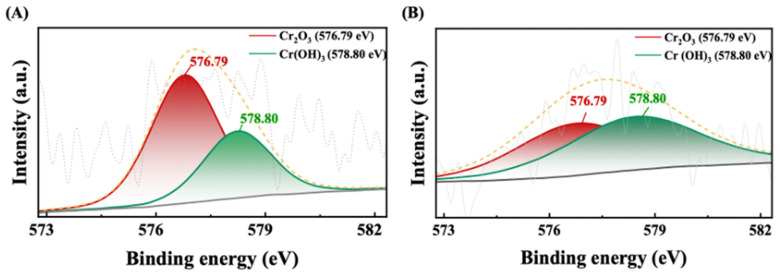
XPS spectra of microalgal cells reveal Cr (III) in both oxide and hydroxide forms. (**A**) The first panel shows cells exposed to 100 ppm of Cr (III). (**B**) The second panel depicts cells treated with 200 ppm of Cr (III).

To gain crucial insights into surface dynamics and electron affinity levels, assisting in the identification of fundamental compositions and chemical states of surface materials, XPS analysis was performed [53]. A detailed examination was performed on freeze-dried biomass exposed to 100 and 200 ppm of Cr (III) to assess potential chemical alterations of Cr (III) within the cellular framework. The results showcased that the peaks perceived in the Cr (III) enriched microalgal biomass at a binding energy (BE) of 576.79 eV were linked to Cr_2_O_3_ (chromium oxide), while those at 578.80 eV predominantly signified the existence of Cr(OH)_3_ (chromium hydroxide) (Figure 3). The atomic percentages for different forms of Cr (III), namely Cr_2_O_3_ and Cr(OH)_3_, were calculated and are presented in Table 1. The findings demonstrated that a considerable proportion of Cr (III) sequestered within the cells exists as Cr_2_O_3_ rather than Cr(OH)_3_. The transformation of Cr_2_O_3_ to Cr(OH)_3_ during bioaccumulation by microalgae encompasses several chemical mechanisms, predominantly influenced by reduction and hydration processes [54]. The reduction of Cr_2_O_3_ to Cr(OH)_3_ may be enhanced under specific chemical catalytic conditions. The presence of trace amounts of potassium and sodium salts significantly accelerates the reduction process, indicating that these salts serve as catalysts in the metamorphosis of Cr_2_O_3_ to Cr(OH)_3_ [55]. Moreover, Cr_2_O_3_ can transform into Cr(OH)_3_ through hydration processes. While the conversion of Cr_2_O_3_ to Cr(OH)_3_ is chiefly governed by reduction and hydration, it is essential to consider the broader environmental context. A thorough grasp of these mechanisms is fundamental for refining bioaccumulation processes and alleviating Cr (III) toxicity in aquatic ecosystems.

### 3.3. Effect of Cr (III) Exposure on Surface Morphology

**Figure 4:**
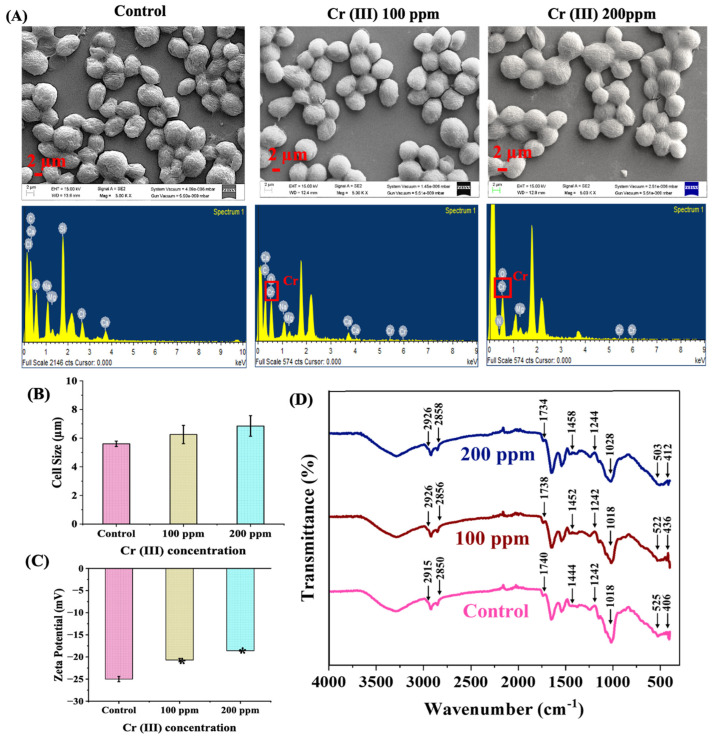
(**A**) FESEM images (top panel) of *C. minutissima* cells exposed to 100 ppm and 200 ppm Cr (III), captured at a 2 μm scale and 5000× magnification, showing cellular morphology. The bottom panel displays EDX micrographs illustrating Cr (III) ion adsorption on cell surfaces under control conditions and at 100 ppm and 200 ppm Cr (III) concentrations. (**B**) Cell size measurements. (**C**) Zeta potential values. (**D**) FTIR spectra of *C. minutissima* cells under control conditions and after exposure to Cr (III) for 12 days. The data denotes the mean ± S.D. from three independent replicates, with *p*-values < 0.05 represented by *, respectively.

Heavy metal exposure has been demonstrated to elicit morphological transformations, which include alterations in size, the advent of anomalies, uneven surface topography, shifts in surface charge, and a myriad of other phenomena. The morphological alterations, paired with a meticulous examination of the localized elemental composition on the cellular surface of *C. minutissima*, were comprehensively explored using SEM alongside EDX (Figure 4A). The SEM visuals revealed minimal surface irregularities on the cells treated with Cr (III), suggesting a potential biosorption of Cr (III) onto the cellular facade. Additionally, the EDX spectra presented distinct peaks linked to Cr (III) in the localized regions of the cell surface, bolstering the notion of Cr (III) adsorption by the microalgal cells. A fascinating shift in cell dimensions unfolded under Cr (III) induced stress, with *C. minutissima*’s average cell size swelling from 5.6 ± 0.19 µm to 6.85 ± 0.72 µm at 200 ppm Cr (III) and 6.25 ± 0.64 µm at 100 ppm Cr (III) after a 12-day incubation (Figure 4B). This phenomenon can be ascribed to the microalgae’s capacity to withstand and metabolize heavy metals, which can precipitate modifications in cellular structure and functionality. Microalgae such as *Chlorella* sp. have demonstrated significant resistance to Cr (III), capable of removing up to 96.5% of Cr (III) from their surroundings. This process entails the adsorption of Cr (III) onto the surface of the cell, followed by its internalization, potentially resulting in an increase in cellular volume due to the buildup of metal ions and corresponding stress responses [56]. 

A notable reduction in the negative charge potential, particularly on the external cellular membrane, with an increase in Cr (III) levels, as shown by zeta potential assessments, further reinforced the binding of trivalent chromium to the cellular surface (Figure 4C). The gathered information showcased a transformation in the surface charge, transitioning from −25 mV in the control cohort to −20.69 mV and −18.59 mV in cells subjected to 100 ppm and 200 ppm of Cr (III), correspondingly. The zeta potential exhibits a decline with increasing concentrations of Cr (III), signifying alterations in surface charge [30]. Variations in zeta potential are fundamental for elucidating the adsorption mechanisms and fine tuning conditions for efficient bioremediation.

Microalgae exhibit a diverse array of functional groups on their cellular surfaces conferring negative charge and facilitate their interaction with metal ions, thereby contributing to detoxification processes. To elucidate the interactions between Cr (III) ions and the functional groups present on the microalgal cellular membrane, FTIR spectroscopy was employed. The FTIR spectra for cultures spiked with Cr (III) exhibit notable shifts and enhanced peak intensities when compared to the control (Figure 4D). For example, the alteration of the peak at 2926 cm^−1^ from 2915 cm^−1^ signifies the asymmetric stretching vibration of methylene (CH_2_), while the shift of the peak from 2850 cm^−1^ in the control to 2858 cm^−1^ in the Cr (III)-exposed cells denotes the symmetric CH_2_ stretching vibration. The upward transition from 2850 cm^−1^ to 2858 cm^−1^ in the FTIR peak towards higher wavelengths suggests alterations in the lipid milieu [5]. Furthermore, this transition is frequently associated with enhanced membrane fluidity and modifications in the cellular membrane architecture. The dip in peak from 1740 cm^−1^ in the control cells to 1734 cm^−1^ in those exposed to Cr (III) signifies the C=O stretching vibrations of ester functional groups. Additionally, the intensified peak and shift from 1444 cm^−1^ to 1458 cm^−1^ represent the bending vibration of CH_2_, initiating disturbances within the hydrocarbon chain alongside changes in protein structure, as this spectral region can also be affected by protein CH_2_ groups [57]. A remarkable peak migration from 1018 cm^−1^ in the control cells to 1028 cm^−1^ in the Cr (III)-treated samples underscores the C-O stretching vibrations of polysaccharides. Similarly, peak shift from 525 cm^−1^ to 503 cm^−1^ confirms interaction of Cr (III) ions with phosphate groups present on the microalgal cell wall. The transition to a lower wavenumber could indicate modifications in phosphate-containing entities (e.g., phospholipids, nucleic acids) [58]. The pronounced change at lower wavenumbers from 406 cm^−1^ to 436 cm^−1^ might reflect a connection with metal–oxygen bonds, often affecting the configuration of metal ions (e.g., magnesium found in chlorophyll) [5,30].

### 3.4. Attenuation of Photosynthetic Efficiency

**Figure 5:**
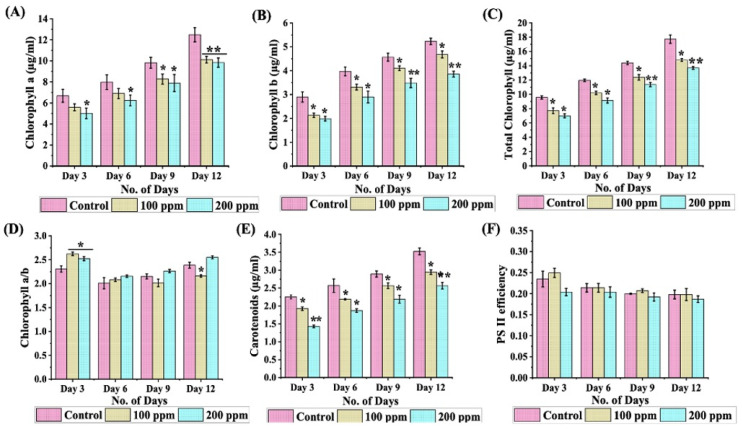
Effects of different Cr (III) concentrations (100 ppm and 200 ppm) on (**A**) chlorophyll a levels; (**B**) chlorophyll b levels; (**C**) total chlorophyll content; (**D**) chlorophyll a/b ratio; (**E**) carotenoid content; and (**F**) PS II efficiency in *C. minutissima*. Measurements were taken every 72 h over a 12-day period. The data denotes the mean ± S.D. from three independent replicates, with *p*-values < 0.05 and 0.01 represented by *, and **.

The photosynthetic ensemble is a uniquely fragile target in the realm of heavy metal-induced stress, with the ability to influence critical physiological and biochemical pathways. The heavy metal exposure induces oxidative stress, leading to the degradation of chlorophyll molecules, which exacerbates the adverse effects on photosynthesis and overall growth [59]. To investigate the ramifications of Cr (III) toxicity, a chronological analysis of photosynthetic efficiency was performed. The results exhibit a notable reduction in chlorophyll a concentration by 24% by day 3, attributable to the deleterious effects of Cr (III) stress (Figure 5A). A corresponding reduction pattern was identified throughout the incubation interval. Notably, chlorophyll a content demonstrated a considerable decline of 18% in cells treated with 100 ppm and 21% in those exposed to 200 ppm Cr (III), in contrast to control cultures by day 12. Furthermore, a similar drift was observed in chlorophyll b levels, demonstrating a 31% reduction on day 3, followed by a gradual resurgence (Figure 5B). As the incubation period wrapped up on day 12, a considerable 26% decline in chlorophyll b levels was documented in cells receiving 200 ppm Cr (III). The data further reveal that total chlorophyll content experienced a parallel reduction, registering declines of 16% and 22% in Cr (III)-exposed groups with 100 and 200 ppm, respectively (Figure 5C). The findings illustrate a coherent chlorophyll a/b ratio, demonstrating that both chlorophyll a and chlorophyll b exhibited parallel diminishing trends in the presence of Cr (III) stress (Figure 5D). The scrutiny of carotenoid quantities in microalgae under the influence of 100 ppm and 200 ppm Cr (III) disclosed a notable upturn in carotenoids across all cultures by day 9, with levels stabilizing for the entirety of the incubation period (Figure 5E). In contrast with the control group, the cells subjected to Cr (III) experienced an approximate 16 to 27% reduction in carotenoid levels by day 12. Further, the ratio of carotenoids to total chlorophyll acts as a metric for assessing the equilibrium between photoprotective processes and light-harvesting efficacy in microalgae [60]. Nonetheless, the findings related to the functionality of photosystem II did not uncover any notable variations between the control group and the Cr exposed cells throughout the incubation phases; however, a subtle reduction was recorded across all cultures by the culmination of the incubation period on day 12 (Figure 5F). 

### 3.5. Analysis of Oxidative Stress and Enzymatic and Non-Enzymatic Antioxidant Molecules

**Figure 6:**
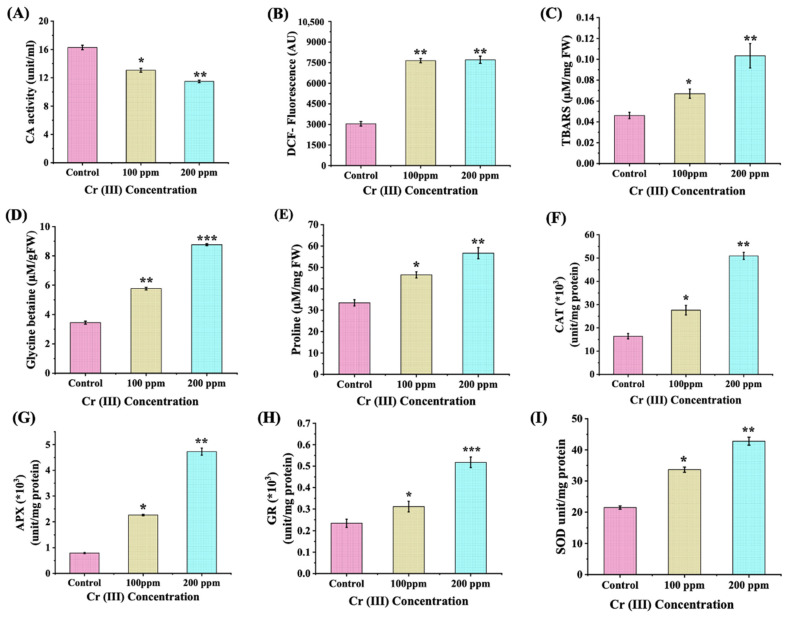
Changes in (**A**) carbonic anhydrase activity; (**B**) total ROS in terms of DCF fluorescence; (**C**) lipid peroxidation in terms of TBARS content; (**D**) total glycine betaine; (**E**) proline content; (**F**) catalase (CAT) activity; (**G**) ascorbate peroxidase (APX) activity; (**H**) glutathione reductase (GR) activity; and (**I**) superoxide dismutase (SOD) activity in *C. minutissima* cultivated in control and Cr (III)-spiked (100 ppm and 200 ppm) media for 12 days. The data denotes the mean ± S.D. from three independent replicates, with *p*-values < 0.05, 0.01 and 0.001 represented by *, **, and ***.

The complex cellular strategies that empower microalgae to navigate Cr (III) stress were scrutinized through a detailed blend of enzymatic and non-enzymatic tests in tandem with the assessment of Cr (III) elicited changes in stress biomolecules and antioxidant pathways. The repercussions of Cr (III) toxicity on algal growth were evaluated by determining carbonic anhydrase (CA) activity (Figure 6A). The infusion of Cr (III) ions into the culture medium resulted in a significant decline (~1.2- to 1.4-fold) in CA activity, indicating a disturbance in the carbonate absorption process and, consequently, impaired photosynthetic performance [35]. These anomalies in the carbon concentration mechanism lead to the synthesis of ROS, which interferes with metal homeostasis and intensifies oxidative stress within the cellular framework. CA activity is primarily contingent upon metal cofactors, and the presence of Cr ions may impede the availability of these crucial cofactors either through competitive inhibition or by disrupting cellular metal homeostasis, thereby causing a decline in CA activity [61]. To exemplify, in *Thalassiosira pseudonana*, Cr (III) ions can act as substitutes for important metal ions like Zn, thereby reducing CA activity and impeding growth [61]. 

The onset of oxidative turmoil triggered by Cr (III) was assessed via the quantification of ROS production, as indicated by the intensity of DCF fluorescence (Figure 6B). Cultures treated with Cr (III) showcased a notable increase of around 2.5 times in ROS generation when juxtaposed with control specimens. This oxidative upheaval was paralleled by a surge in lipid peroxidation levels across all experimental samples, unveiling a ~1.4- to 2.2-fold increase in TBARS content (Figure 6C). In the wake of ROS toxicity, microalgae craft a tapestry of antioxidant compounds coupled with diminutive osmolytes, including proline and glycine betaine (GB) [10]. The findings showed a ~1-fold rise in proline levels (Figure 6D) along with a notable 1.7- to 2.5-fold escalation in GB levels in cells facing Cr (III) exposure (Figure 6E).

Heightened oxidative stress, instigated by heavy metal exposure, prompts the gradual activation of inherent enzymatic antioxidant mechanisms in microalgae, serving as a crucial adaptive strategy to mitigate stress. The efficacy of this enzymatic reaction in *C. minutissima* subjected to Cr (III) was assessed by measuring the activities of antioxidant enzymes such as CAT, APX, SOD, and GR. The findings revealed a remarkable enhanced CAT activity by 1.7- to 3-fold (Figure 6F), succeeded by an impressive 3- to 6-fold increase in APX activity (Figure 6G), alongside a substantial 1.3- to 2.2-fold rise in GR activity (Figure 6H) in cells treated with Cr (III) (100 ppm and 200 ppm, respectively). In a similar vein, SOD activity in cells subjected to Cr (III) showcased a striking 1.6- and 2-fold increase at concentrations of 100 ppm and 200 ppm, respectively (Figure 6I). The noted changes in enzymatic antioxidant activities imply the engagement of a resilient defense mechanism within the cell, which counteracts on the oxidative stress and supports the survival of *C. minutissima* in the presence of Cr (III) toxicity.

### 3.6. Analysis of Temporal Based Biochemical Response

**Figure 7:**
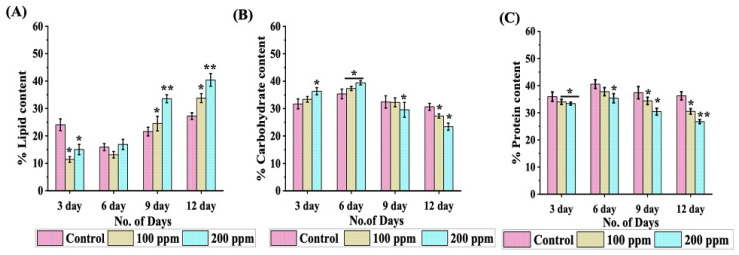
Time-based analysis of (**A**) lipid content; (**B**) carbohydrate content; and (**C**) protein content in *C. minutissima* biomass under control conditions and with Cr (III) exposure at 100 ppm and 200 ppm, over a 12-day incubation period. The data denotes the mean ± S.D. from three independent replicates, with *p*-values < 0.05 and 0.01 represented by * and **.

The discernible decline in cellular division and photosynthetic efficacy in Cr-treated microalgal cells indicates compromised carbon concentration regulation mechanism. Microalgae reveal a fascinating ability to shift carbon dynamics towards the formation of reserve compounds, such as lipids and carbohydrates, facilitating their adaptability and endurance in various stress-filled habitats, particularly those plagued by heavy metal toxicity. A thorough 12-day temporal analysis was performed to elucidate biochemical transformations induced by Cr (III) toxicity in *Chlorella minutissima* (Figure 7). Data indicated that cultures facing the peak Cr (III) concentrations, i.e., 200 ppm, showcased a remarkable 1.5-fold surge in total lipid accumulation by day 9, eventually plateauing at nearly 40% lipid content by day 12 (Figure 7A). During this period, cell division slows as the culture progresses towards the stationary phase, coinciding with significant resource limitations. Additionally, the rate of lipid accumulation decreases as metabolic processes shift their focus from growth to maintenance.

Further, quantitative evaluation of carbohydrate accumulation demonstrated that exposure to Cr (III) in *C. minutissima* led to a noteworthy increase in total carbohydrate levels, reaching peak values of approximately 37% and 40% in cells subjected to 100 ppm and 200 ppm Cr (III), respectively, by day 6 (Figure 7B). A comparable pattern was noted in control cells, wherein the maximum carbohydrate content (~35%) was similarly recorded on day 6. By the end of day 12, cells spiked with Cr (III) displayed a striking ~1.3 fold (~22%) dip in carbohydrate levels compared to the control group (~30%). 

A thorough investigation of the total protein concentration demonstrated a noteworthy rise in Cr (III) treated cells during the introductory adaptive phase, which was subsequently followed by an approximate 1.4-fold reduction by the culmination of day 12 (Figure 7C). The notable uptick in protein abundance recorded in cells exposed to Cr (III) by day 6 indicates the engagement of metal-binding agents or chelating factors that limit the presence of free ions [62]. This initial elevation in protein content signifies the organism’s adaptive mechanisms aimed at alleviating the detrimental impacts of heavy metals. An analogous elevation in protein concentrations was noted in *Monoraphidium* cells encountering heavy metal stress [62]. Nevertheless, heavy metal ions can adhere to proteins and nucleic acids, causing disruptions in enzymatic actions and crucial cellular functions like DNA replication, transcription, and translation, thereby impeding protein synthesis [63]. The pronounced reduction in protein content recorded during the protracted exposure of 12 days may be ascribed to these interactions, which compromise cellular functions and lead to diminished protein synthesis [63]. In addition, the drop in photosynthetic efficiency under Cr (III) stress curtails the carbon supply, thus intensifying the reduction in protein levels [64]. As cultures transition towards the stationary phase, a slight dip in protein content is a common physiological response shown by microalgae due to their internal cellular metabolism. The dynamic biochemical changes uncovered in this study demonstrate that under Cr (III) stress, *C. minutissima* initially accumulates carbohydrates to mitigate stress, later steering its carbon metabolism towards lipid synthesis as it progresses through the exponential growth phase.

### 3.7. ^1^H NMR-Based Lipid Profiling of Cr (III)-Exposed C. minutissima

**Figure 8:**
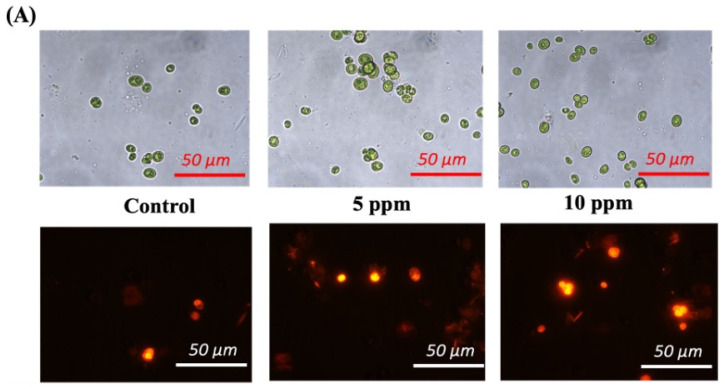

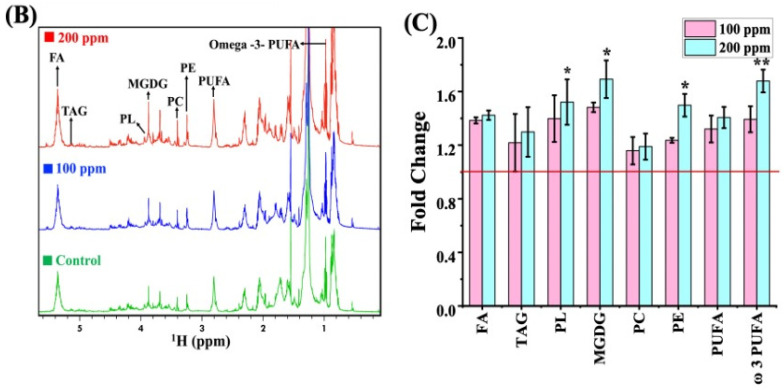
(**A**) Bright field microscopy visuals (upper section) of *C. minutissima* after 12 days of growth (scale: 50 μm) and fluorescence microscopy visuals (lower section) of *C. minutissima* dyed with Nile red (scale: 50 μm). (**B**) Exemplary ^1^H NMR spectra showcasing the overall lipid compositions of *C. minutissima* under standard conditions and after exposure to Cr (III) at concentrations of 100 ppm and 200 ppm. (**C**) Analysis of fold changes revealing shifts in various lipid categories. Within this context, the current study explores the integration of molecular mechanisms linked to Cr (III) tolerance, the rerouting of carbon metabolism triggered by Cr (III) towards lipid synthesis, and valuable by-products in the heavy metal-adaptive green microalga *C. minutissima*. Abbreviations: FA—fatty acid residue; TAG—triacylglyceride; PL—total phospholipid; MGDG—monogalactosyl diacylglycerol; PC—phosphatidylcholine; PE—phosphatidylethanolamine; PUFA—polyunsaturated fatty acid; omega-3-PUFA/ω3 PUFA—omega 3 polyunsaturated fatty acid. The data denotes the mean ± S.D. from three independent replicates, with *p*-values < 0.05 and 0.01 represented by * and **.

A multifarious configuration of lipid structures is essential for sustaining membrane integrity, facilitating photosynthesis, reserving carbon and energy, refining signaling pathways, and ensuring robustness against stress, thus occupying a critical position in the overall physiological and metabolic dynamics of microalgae. Nile red staining unveiled vividly bright yellow lipid droplets within cells treated with Cr (III) as compared to controls, indicating the enhanced lipid production under stress (Figure 8A). In light of this, the lipid profiles of microalgal cells undergoing Cr (III) stress were analyzed through the lens of ^1^H NMR spectroscopy. The ^1^H NMR spectra of lipids from both control and Cr (III)-treated cells illustrated a captivating collection of fatty acid moieties (FAs), triacylglycerides (TAGs), phospholipids (PLs), monogalactosyl diacylglycerol (MGDG), phosphatidylcholine (PC), phosphatidylethanolamine (PE), polyunsaturated fatty acids (PUFAs), and omega-3-PUFA (Figure 8B). Compositional scrutiny revealed that Cr (III) stress triggered an enhancement in production across all lipid classes, barring PC (Figure 8C). In particular, cultures exposed to 200 ppm Cr (III) exhibited the most pronounced changes (~1.7-fold) in omega-3-PUFA and MGDG, followed by notable shifts in PL and PE (~1.5-fold). Fatty acid moieties and PUFA showed ~1.4-fold fluctuations, while TAG concentrations altered by ~1.3-fold. These extensive transformations in structural and storage lipid levels suggest a meticulous orchestration of fatty acid creation and reconfiguration within algal cells as a strategic comeback to mitigate Cr (III) stress. 

### 3.8. FAME Profiling and Assessment of Biodiesel Properties

**Figure 9:**
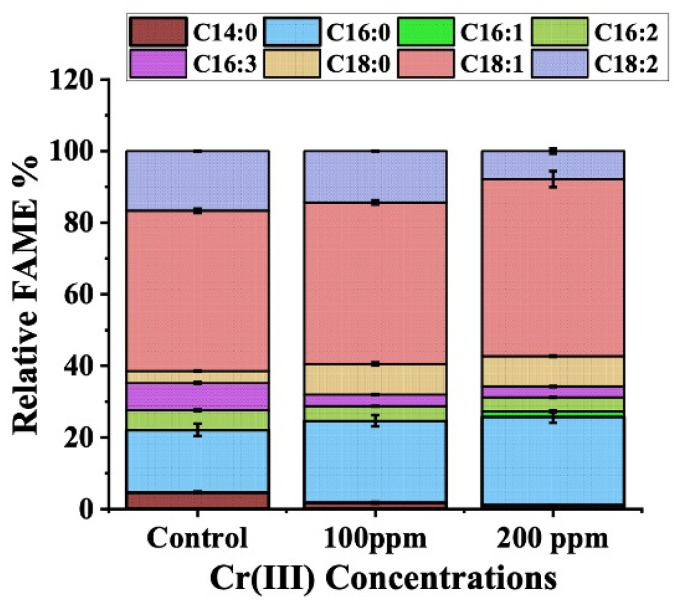
FAME profile of control and Cr (III) spiked lyophilized biomass after 12 days of incubation.

The lipid generated by *C. minutissima* under the impact of Cr (III) stress was scrutinized for its fatty acid makeup. The resulting data were utilized to assess the vehicular eminence and physical characteristics of the biodiesel generated, in comparison with benchmarks established by the American Society for Testing and Materials (ASTM D6751-02) [65] and European standards (EN 14214) [10,66,67]. The compositional landscape of *C. minutissima* in the absence of Cr (III) stress revealed a dominance of oleic acid (C18:1), palmitic acid (C16:0), and linoleic acid (C18:2), alongside hexadecatrienoic acid (C16:3), hexadecadienoic acid (C16:2), myristic acid (C14:0), and stearic acid (C18:0) (Figure 9). Cr (III) stress notably enhanced the levels of saturated fatty acids (SFAs), such as C16:0 and C18:0, by approximately 1.8 times. A parallel escalation (about 1.5 times) in monounsaturated fatty acids (MUFAs), primarily C18:1, was documented. Conversely, there was a significant reduction of about 1.5 times in polyunsaturated fatty acids (PUFAs), including C16:2, C16:3, and C18:2, in cultures exposed to Cr (III). These variations in the FAME composition led to a noteworthy decline in the unsaturated to saturated fatty acid (UFA/SFA) ratio, plummeting from 2.92 in the control group to 2.0 and 1.86 in cultures subjected to 100 ppm and 200 ppm Cr (III), correspondingly. This metamorphosis reflects a shift in membrane resilience and fluidity, epitomizing a keen adaptation to Cr (III) toxicity.

To evaluate the relevance of lipids sourced from *C. minutissima* exposed to Cr (III) stress for biodiesel synthesis, vital physical characteristics such as saponification value (SV), iodine value (IV), cetane number (CN), degree of unsaturation (DU), long-chain saturation factor (LCSF), high heating value (HHV), cold flow plugging property (CFFP), and oxidative stability (OS) were derived using their empirical formula (Table 2). The SV serves as a direct indicator of ester linkages, with the results falling within the desirable range of 220–240 mg/KOH, a vital signifier for well-balanced fatty acid profiles essential for efficient combustion and energy yield in biodiesel [68]. The determined SV values (145–168 mg/KOH) indicate an adequate average molecular weight of the fatty acids, critical for ensuring suitable viscosity and flow dynamics of biodiesel. Cr (III) stress inflicted an oxidative damage upon microalgal cells, depleting PUFA levels and consequently bolstering the oxidative stability (OS) of the bio-oil, which peaked at 21 h in cultures exposed to 200 ppm Cr (III) [69]. The decline in the UFA/SFA ratio due to Cr (III) stress markedly influences both OS and CFFP. The enhanced OS of the bio-oil may be ascribed to the elevated levels of SFAs, which exhibit greater resistance to oxidation when compared to UFAs [70]. This increased SFA concentration endows the bio-oil with superior thermal and oxidative endurance, thereby prolonging its shelf life, augmenting fuel stability, and mitigating degradation risks. Conversely, the elevated SFA ratio detrimentally affects the CFFP, as SFAs tend to solidify at diminished temperatures. This phenomenon leads to impaired low-temperature performance, potential challenges in engine ignition in frigid climates, and heightened fuel viscosity [71]. While the augmented OS is beneficial for storage and stability, the compromised CFPP constrains the biodiesel’s applicability in colder settings. In addition to enhanced OS, the high heating value (HHV) of the algal oil remained firmly within the designated range of 41–42 MJ/kg. Indeed, observed modifications in the automotive properties of transesterified biolipid obtained from Cr (III)-spiked biomass emphasize the potential of *C. minutissima* as an essential participant in biorefinery initiatives that offer a comprehensive strategy for Cr (III) detoxification and the production of valuable biodiesel.

### 3.9. Compositional Analysis of EPS Secreted by C. minutissima Under Cr (III) Stress

**Figure 10:**
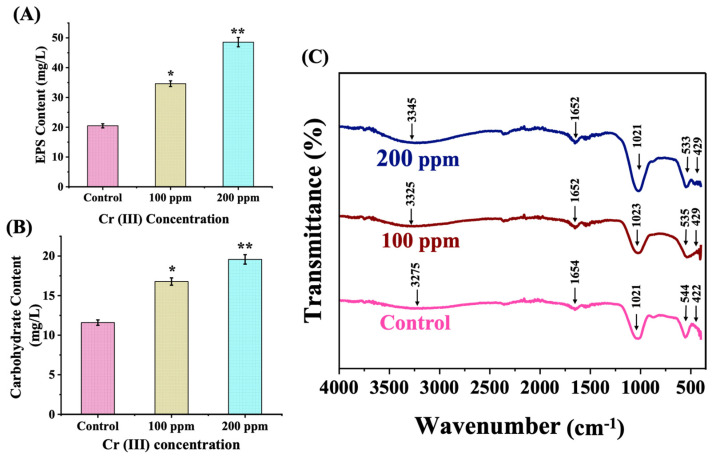
(**A**) Total soluble extracellular polysaccharide content (EPS). (**B**) Total carbohydrate content in soluble EPS. (**C**) FTIR spectra of lyophilized EPS powder from supernatant of control and Cr (III)-spiked microalgal cultures.The data denotes the mean ± S.D. from three independent replicates, with *p*-values < 0.05 and 0.01 represented by * and **, respectively.

In conjunction with the internal tuning of carbon flux and the modulation of antioxidant enzymes, the release of extracellular polysaccharides (EPSs) constitutes a pioneering response to stress in microalgae, enhancing the chelation and detoxification of Cr (III). In addition to their role in heavy metal detoxification, EPSs unveil a wealth of biotechnological opportunities, spanning environmental rejuvenation and medical innovations [29]. Assessment of soluble EPSs from *C. minutissima* indicated a substantial elevation (~1.7- to 2.5-fold) under Cr (III) stress (Figure 10A), with carbohydrate amounts in the Cr (III)-exposed cohort reflecting a considerable ~1.5- to 1.8-fold increase compared to the control (Figure 10B). Reflecting these insights, a variety of microalgal species have been noted to exhibit increased EPS synthesis when exposed to the stressors of heavy metals (Table S5). The FTIR scrutiny of lyophilized EPSs revealed distinct vibrational bands with enhanced intensity within the 900–1200 cm^−1^ spectrum, suggesting the presence of phosphate sugars, acidic sugars, and glycosidic linkages in both control and Cr (III)-exposed cultures (Figure 10C). The vibration band at 1021 cm^−1^ was attributed to the C–O–C bonds of polysaccharides [72]. The peak at 3275 cm^−1^ in the control displays a shift towards a higher wavelength to 3345 cm^−1^, represents -OH stretching, and is generally broad with U shape. This FTIR analysis illustrated that the vital vibrational frequencies of polysaccharides remain intact in both the control and Cr (III)-treated cultures, implying that the integrity of EPS persisted reliably under Cr stress. EPSs of such an exquisite quality, cultivated in environments tainted by Cr (III), can be artfully utilized for innovative biotechnological applications, such as refinement of wastewater.

## 4. Discussion

**Figure 11:**
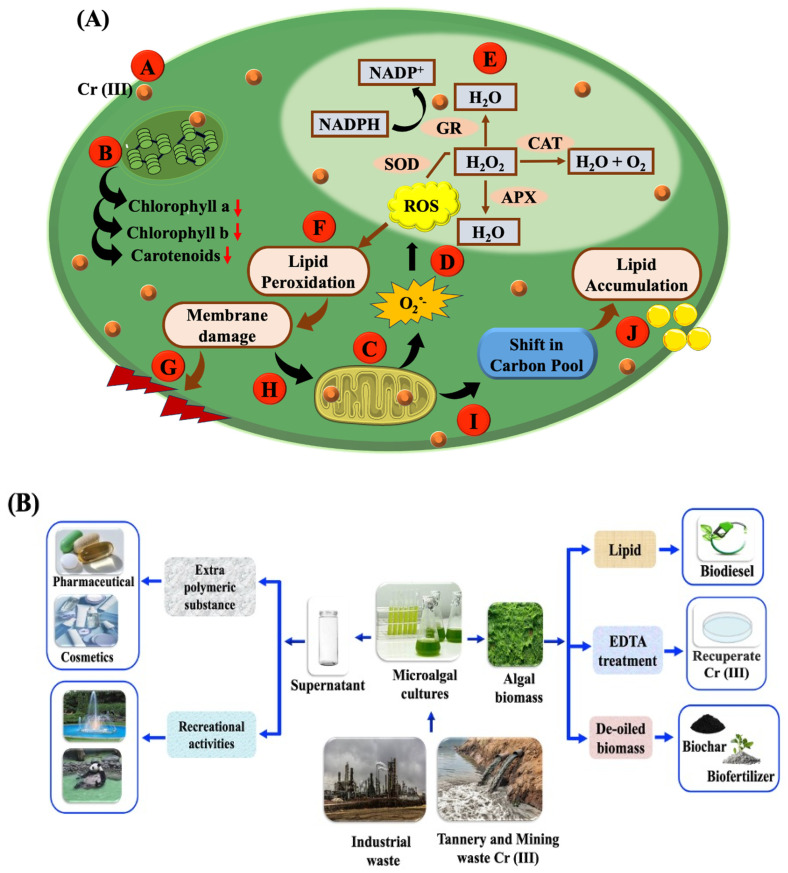
(**A**) A schematic representation depicting the tolerance mechanism adopted by *C. minutissima* to survive under Cr (III) stress. Various steps involved in survival mechanism adopted by *C. minutissima* involves: (A). Interaction of Cr (III) ions with the functional groups present on algal cellular surface; (B) Impaired photosynthesis machinery due to Cr (III) stress; (C) Disruption of mitochondrial metabolic activities; (D) Generation of oxidative stress within the cell; (E) Activation of antioxidant machinery as defense mechanism; (F) Lipid peroxidation induced due to elevated levels of ROS; (G) Disruption of cellular membrane/membrane lipids; (H–I) in response *C. minutissima* shift carbon flux towards synthesis of energy reservoirs; (J) increased lipid production. (**B**) A conceptual illustration portraying an environmentally friendly biorefinery design that harnesses Cr (III) resistant microalgae for the purpose of environmental remediation while concurrently facilitating the production of biodiesel and an array of high-value substances like exopolysaccharides (EPS).

The astonishing knack of microalgal cells to amass energy reserves in response to challenges stands out as a captivating characteristic that offers an eco-friendly approach intertwining bioremediation, biofuel production, and synthesis of valuable products within a biorefinery framework. Within this conceptual framework, the present research uncovers the elaborate amalgamation of molecular processes related to Cr (III) resistance, as well as the diversion of carbon metabolism initiated by Cr (III), resulting in lipid generation and advantageous value-added products in the heavy metal-adapted green microalga *C. minutissima*. The analysis explores the shifts in lipid dynamics, extracellular polysaccharide (EPS) levels, and the associated antioxidant defense mechanisms induced by Cr (III) to uncover the vital strategies for stress adaptation adopted by *C. minutissima* (Figure 11).

### 4.1. Insights into the Cr (III) Detoxification and Lipid Accumulation Dynamics

Indeed, trivalent chromium is a hazardous heavy metal present in our ecological system, sourced more prevalently due to various anthropogenic activities while at the same time mother nature “Earth” is facing a huge crisis in the renewable energy front. These predicaments paved ways for green energy fuel maturity along with the latitude of heavy metal mitigation in a unified biorefinery structure. In this study, *C. minutissima* was able to withstand Cr (III) toxicity with an exceptional removal efficiency of 92–98% with 200 and 100 ppm Cr (III) concentrations, respectively (Figure 2A). Previous studies have documented Cr (III) remediation efforts; however, our research utilized significantly higher concentrations of trivalent chromium, achieving approximately 226 mg of mitigated Cr (III), indicating *C. minutissima* as a Cr (III) hyper-resilient strain (Table S4). Microalgal cells activate a rich tapestry of tolerance mechanisms in the quest for heavy metal remediation, incorporating both bioadsorption and bioaccumulation pathways. In this study, *C. minutissima* demonstrated significant chromium accumulation, with approximately 145 ± 2.07 mg/g of Cr (III) retained within the cells and an additional 78 ± 2.12 mg/g adsorbed onto the cell surface (Figure 2B,C). Additionally, previous studies have highlighted the Cr (III) bioremediation capabilities of various microalgae such as *Chlorella thermophia*, *Chlorella minutissima* [56], *Chlamydomonas* sp., [73], *Auxenochlorella pyrenoidosa*, *Scenedesmus* sp., [74], *Dunaliella* sp. AL1 [75], *Spirulina* sp. [76], and *Chlorella pyrenoidosa* [77]. 

The interplay of electrostatic forces between the anionic cell membrane and the cationic Cr (III) ions is crucial in amplifying metabolic activities, including membrane translocation and cellular assimilation. The decline in negative charge potential from −25 mV in the control to −18.59 mV in the Cr (III)spiked group illustrates *C. minutissima*’s ability to capture, sequester, and eradicate Cr (III) from wastewater, positioning them as outstanding candidates for bioremediation of chromium pollution (Figure 4C). Moreover, microalgae are endowed with various functional groups on their cellular surfaces, such as carboxyl, hydroxyl, and amino groups, which augment the binding and incorporation of heavy metals such as Cr (III) [78]. The engagement of Cr (III) ions with the functional groups located on the cell surface is highlighted through the acquired FTIR data, emphasizing significant peak displacements that represent both asymmetric and symmetric CH_2_ stretching, along with C=O and C-O stretching. Moreover, the downward shift from 525 cm^−1^ to 503 cm^−1^ distinctly points to the interaction between phosphate groups and Cr (III) ions, indicating a transformation in the phospholipid structure (Figure 4D) [58].

In microalgae, the presence of Cr (III) has been shown to curtail photosynthetic prowess, chiefly due to the interference with the photosynthetic electron transport chain instigated by Cr (III) [79,80]. Cr (III) ions can replace magnesium ions in chlorophyll molecules, which are essential for capturing light energy during photosynthesis. This alteration disrupts the structural framework of chlorophyll, culminating in lower photosynthetic effectiveness and hindered carbon fixation [81]. Cr (III) impairs the synthesis of chlorophyll by obstructing the integration of magnesium into protoporphyrin IX and competing with vital metal ions that are integral to the chlorophyll structure [82]. Consequently, this disruption adversely affects the enzymatic pathways involved in chlorophyll biosynthesis. A decrease in chlorophyll concentration directly constrains the microalga’s capacity to efficiently capture light, resulting in a reduction of energy generation [83]. This, in turn, impedes cellular proliferation and division, thereby affecting overall biomass accumulation. In line with these arguments, our findings reveal a downturn in photosynthetic efficiency, with chlorophyll a declining by nearly 18–21% and chlorophyll b dropping by ~26%, thus altering the comprehensive pigment yield (Figure 5A,B). A decrease in chlorophyll content typically reflects a decline in the ability of the microalgae to capture light for photosynthesis, which may lead to reduced photosynthetic efficiency. In our study, the observed reduction in chlorophyll content serves as an indirect indicator of impaired photosynthetic activity under Cr (III) stress. Consistent with these findings, heavy metal supplementation in the growth medium resulted in a reduction in pigment synthesis in species such as *C. pyrenoidosa*, *S. acutus*, *S. quadricauda*, *C. minutissima*, *C. vulgaris*, and *Coccomyxa* sp., among others [69,84,85,86]. Carotenoids, the vibrant hydrophobic pigments, are key architects in the dance of light absorption and photoprotection within microalgal realms [87]. These pigments function as buffers against oxidative stress by quelling reactive oxygen species, thus dodging prospective cellular dysfunctions [88]. In the context of exposure to Cr (III), the heavy metal can induce oxidative stress, resulting in the degradation of chlorophyll molecules and impairment of photosystem II (PSII) [89]. This deterioration diminishes light-harvesting capability and electron transport efficiency, further exacerbating the negative effects on photosynthesis and growth. Therefore, the reduction in carotenoid levels (~16–27%) highlighted in our analysis uncovers an elevation in oxidative stress fostered by Cr (III) (Figure 5E). Microalgae exhibit remarkable resilience strategies to combat heavy metal challenges; as an example, the engagement of antioxidant mechanisms in *Scenedesmus obliquus* facing heavy metal adversity significantly aids in preserving photosystem II functionality by alleviating oxidative impairment through strategies such as non-photochemical quenching and managed energy dissipation [90]. The unwavering ratios of chlorophyll a/b and carotenoid/total chlorophyll demonstrate that *C. minutissima* has the prowess to respond to chromium-induced stress by fostering a stable equilibrium in pigment biosynthesis, thereby fortifying the photosynthetic apparatus against oxidative injury.

Cr (III) sparks the generation of reactive oxygen species (ROS) like superoxide anions (O_2_^−^), hydrogen peroxide (H_2_O_2_), and hydroxyl radicals (.OH) [91]. Metal ions can interfere with cellular frameworks, inducing electron leakage in the electron transport chains of chloroplasts and mitochondria. Moreover, the stress from heavy metals activates NADPH oxidase, prompting swift electron transfer to molecular oxygen, which results in a notable increase in superoxide radicals [92]. This process intensifies the overall pathways for ROS production. Indeed, the rise in ROS triggers the initiation of oxidative stress pathways such as lipid peroxidation, protein oxidation, DNA damage, etc. [93]. To combat heightened oxidative stress, microalgae unleash a sophisticated arsenal of both enzymatic and non-enzymatic antioxidants. In this study, the amplified presence of osmolytes such as proline and GB illustrates their remarkable adaptive responses targeted at countering the adverse ramifications of Cr (III) (Figure 6D,E). These compounds play the role of osmotic defenders, stabilizing proteins and membranes, neutralizing free radicals, and thereby enriching the stress resilience of microalgae. Moreover, the heightened synthesis of antioxidant enzymes like SOD, CAT, APX, and GR showcases the resilience of our microalga, effectively countering the detrimental impacts of ROS in a cascading manner (Figure 6F–I). SOD serves as the frontline guardian against ROS by transforming superoxide radicals into hydrogen peroxide and molecular oxygen. For example, SOD activity in *Gracilariopsis lemaneiformis* is enhanced to maintain ROS equilibrium under copper-induced stress [94]. CAT efficiently converts hydrogen peroxide, a byproduct of SOD activity, into water and oxygen, effectively shielding cells from potential damage [95]. In *Coccomyxa onubensis*, CAT activity has seen a significant jump when faced with heavy metal stress, accentuating its vital contribution to the antioxidant defense system [96]. APX leverages ascorbate as a foundational element to neutralize hydrogen peroxide, performing an indispensable function in the ascorbate–glutathione cycle, which is critical for the maintenance of cellular redox stability [95]. GR, subsequently, reconstructs reduced glutathione from its oxidized version, effectively sustaining the cycle and strengthening the cell’s defenses against oxidative stress [96]. The enzymatic functions of SOD, CAT, APX, and GR are interconnected, collaborating in a synergistic fashion to remove ROS and diminish oxidative stress within the cellular domain (Figure 11A).

To withstand Cr (III) toxicity, microalgae tweak their redox dynamics by rerouting carbon flux towards the synthesis of stress-mitigating metabolites like lipids and carbohydrates. The observed initial increase (~1.2-fold) followed by decline (~1.42-fold) in carbohydrate content in our study can be associated with the fact that during the formative period of exponential growth, carbohydrates serve as the key outcomes of photosynthesis, rapidly building up within microalgal cells and energizing crucial metabolic activities such as DNA replication, nuclear division, and cytokinesis (Figure 7B) [97]. As the growth phase progresses into the latter part of the exponential stage, the breakdown of carbohydrates occurs, steering carbon frameworks towards the synthesis of lipids [10]. This reduction in overall carbohydrate levels under Cr (III) stress may be attributed to the likely suppression of carbon metabolism, potentially stemming from the interaction of Cr (III) ions with the active sites of essential enzymes, including ribulose-1,5-bisphosphate carboxylase [98]. Prolonged exposure to heavy metals can inhibit key enzymes involved in carbohydrate metabolism. This is supported by findings that heavy metals significantly decrease the activities of enzymes like β-glucosidase and invertase, which are crucial for carbohydrate breakdown and utilization [99].

Amidst Cr (III) stress, *C. minutissima* exhibits augmented lipid accumulation, indicative of enhanced metabolic rates and potential enlargement of cellular size paving the way for biofuel advancement [56]. According to the findings of this research, cells infused with 200 ppm Cr (III) exhibit a striking lipid buildup of 40% by day 12 (Figure 7A). In a similar manner, Liu et al. (2024) documented a considerable rise in lipid content (25%) within *Chlorella pyrenoidosa* under 5 ppm Cr (III) stress, ascribed to the upregulation of antioxidant systems and pivotal enzymes implicated in lipid biosynthesis, such as acetyl-CoA carboxylase (ACC), which were elevated in response to Cr (III) exposure [77]. Further, exposure to chromate ions results in significant changes in membrane lipid composition including decrease in phosphatidylglycerols (PGs) and sulfoquinovosyldiacylglycerols (SQDGs), which suggests a reallocation of resources towards other lipid classes to maintain cellular integrity under stress [100]. In alignment with these observations, numerous microalgal taxa have been documented to demonstrate enhanced lipid accumulation when subjected to heavy metal-induced stress (Table S6).

The dynamic shifts in neutral and charged lipid concentrations are crucial for stress acclimatization, as NMR lipid profiling revealed notable increases in omega-3-PUFA and other lipids, confirming the reorganization of lipid reserves triggered by Cr (III) (Figure 8C). Prior investigations have established that microalgae such as *Chlorella* sp. and *Chlamydomonas* sp. enhance lipid accumulation, encompassing omega-3 PUFAs, in response to various stress conditions [101]. The surge in polar lipid levels reflects a strategic maneuver by microalgal cells to combat Cr (III) toxicity while maintaining membrane-related cellular functions. In response to heavy metal stress, microalgae demonstrate striking modifications in their lipid profiles, especially in polar lipids, comprising phospholipids and glycolipids. Such alterations are crucial for upholding membrane integrity and functionality, which are vital for the survival of cells during stressful conditions [102]. Indeed, Cr (III)-driven stress incites both catabolic and anabolic processes in *C. minutissima*, undermining its structural cohesion, sapping energy reserves, and transforming signaling activities

### 4.2. Sustainable Biorefinery Approach Using C. minutissima Under Cr (III) Stress: Socio-Economic Perspectives

In view of the broad adoption of Cr (III) in numerous domains like automotive, metallurgy, textiles, dyes, and leather, enforcing a restriction on its application is unrealistic, despite the health and ecological hazards associated to prolonged exposure. However, microalgae exhibiting resilience against harmful heavy metals like Cr (III) offer a promising pathway to tackle the interconnected dilemmas of energy production, environmental degradation, and economic sustainability. This study unveils an eco-friendly biorefinery framework harnessing *C. minutissima*, facilitating bioremediation of 92% Cr (III) (200 ppm) while at the same time yielding lipid-abundant biomass and extracellular polysaccharides (EPSs). Withstanding the perils of Cr (III) toxicity, *C. minutissima* skillfully transforms its carbon flow to craft high-grade biodiesel tailored for transportation. In addition, the exquisite EPSs (~2.5-fold) produced by this microalga clear the path for countless biotechnological strides, extending from ecological healing to pioneering medical discoveries [35]. The unique flocculation features of these EPSs can be tapped into for wastewater management, advancing sustainable environmental solutions [36]. Additionally, EPSs are highly valued in various chemical and biotechnological industries due to their versatile bioactive properties’ potential wellness benefits [103,104]. The multifaceted design of EPS formulations elevates their economic practicality by granting the ability to craft specific applications across various domains (Figure 11B) [103].

Furthermore, Cr (III) can be proficiently retrieved from aqueous solutions by employing microalgal biomass paired with chelating materials such as ethylenediaminetetraacetic acid (EDTA) [35]. The Cr (III)-enriched water can be delightfully utilized for recreational pursuits, while the de-fatted biomass serves as a versatile resource for biofertilizers, animal feed, and biochar, among other applications. Amidst the lipid extraction process, a considerable segment of the Cr (III) stays embedded in the methanolic extract, thereby safeguarding the purity of the biodiesel and the de-oiled biomass [105]. Recent studies have illuminated the environmental viability and economic practicality of integrating wastewater treatment with the creation of biofuels and bioproducts via a microalgal-focused biorefinery model [106,107]. Predictions reveal that generating 10,000 tons of algal biomass (up to 30% lipids) could incur expenses nearing 1.4–1.8 USD/L, starkly differing from the existing market rate of 0.79 USD/L for mineral diesel [108]. Moreover, the techno-economic analysis revealed that the baseline selling price of biodiesel can be significantly reduced (up to 0.98–1.18 USD/L) by coupling it with wastewater treatment [109]. The scrutiny of these extraordinary algal strains not only identifies them as powerful collaborators in bioremediation but also paves a promising route towards biofuels produced from algae. As a whole, the primary revelations from this study accentuate the potential for a pragmatic and eco-sensitive solution to the socio-economic dilemmas related to algal biorefineries.

## 5. Conclusions

This study revealed the dynamic, time-oriented adaptive responses of *C. minutissima* under Cr (III) stress. This remarkable hyperaccumulator strain demonstrated an extraordinary capacity to eliminate 92–98% of Cr (III), with an IC_50_ value of 200 ppm. The presence of Cr (III) ions in cellular architectures can wreak havoc on crucial metabolic processes, ushering in oxidative stress through a surge of ROS, which leads to heightened lipid peroxidation and obstructed pigment production. The rise in oxidative stress triggered the mobilization of both enzymatic (CAT, APX, SOD, and GR) and non-enzymatic (proline and GB) antioxidant systems in a coordinated manner, representing a steadfast tactic for addressing stress. To alleviate the adverse effects of Cr (III)-induced toxicity, microalgal cells ingeniously reshape their redox milieu by shifting carbon flow towards the formation of stress-mitigating compounds such as lipids and EPSs. In addition, the FAME profile and properties of the biodiesel produced are in accordance with American and European standards, accentuating the potential of *C. minutissima* for the generation of biodiesel in a sustainable and economically advantageous manner. Collectively, these biochemical revelations offer profound mechanistic understanding of the detoxification process of Cr (III) by *C. minutissima* and the generation of commercially valuable products like biodiesel and extracellular polysaccharides. Indeed, exploring such precious algal strains collectively lays the groundwork for an eco-friendly and economically viable solution for effective remediation and biofuel production via a biorefinery approach. 

## Figures and Tables

**Table 1:** Atomic composition evaluation of Cr_2_O_3_ and Cr(OH)_3_ in *C. minutissima* subjected to Cr (III).

Initial Cr (III) Concentration	Atomic Percentage of Cr_2_O_3_	Atomic Percentage of Cr(OH)_3_
100 ppm	65.6	34.3
200 ppm	63	36.9

**Table 2:** Exploratory evaluations of the biodiesel characteristics of FAME derived from *C. minutissima* cultivated in varying levels of chromium (III) juxtaposed with ASTM D6751, EN 14214 fuel specifications, and methyl esters from plant oils.

Physical Properties	Standard Fuel Parameter		Control	Cr (III)-Exposed Cells		Plant Oil Methyl Esters	
	ASTM (D6751-02)	EN (14214)	BBM	100 ppm	200 ppm	JME	PME
Saponification value (mg KOH)	-	-	128	145	168	96	49
Iodine value (g I_2_/100 g)	-	120 max	68	78	79	-	-
Cetane number	47 min	-	71	64	58	54	61
Degree of unsaturation (% weight)	-	-	65	62	62	-	-
Long-chain saturation factor (% weight)	-	-	2.	5	6	-	-
High heating value (MJ/kg)	-	-	43	42	41	-	-
Cold flow plugging property (°C)	-	≤5/≤−20	−9.2	−0.75	2.3	−2	13
Oxidative stability (h)	-	>6	13	14	21	3.9	16.5

## Data Availability

The data presented in this study are available on request.

## Supplementary Materials

The following supporting information can be downloaded at: https://www.mdpi.com/article/10.3390/cells13242047/s1. Supplementary Figure S1. Cultures of *Chlorella minutissima* cultivated in control (BBM) and at 100 and 200 ppm of Cr (III) spiked in BBM for 12 days. Supplementary Table S1. Composition of Bold’s basal media (BBM) and micronutrient solution. Supplementary Table S2. Composition of micronutrient solution. Supplementary Table S3. Empirical formulas for calculating biodiesel physical properties. Supplementary Table S4. Comparative analysis of IC50 values and Cr (III) removal efficiency (%) by different microalgae. Supplementary Table S5. A comprehensive examination of EPS biosynthesis and its carbohydrate content in the context of heavy metal exposure. Supplementary Table S6. Summary of lipid accumulation in response to different heavy metal stress in microalgal species. References [110,111,112,113,114,115,116,117,118,119,120,121,122,123] are included in Supplementary Materials file.
