# Supplementary material for: A Biorefinery Approach Integrating Lipid and EPS Augmentation Along with Cr (III) Mitigation by Chlorella minutissima
Source: Cells. 2024 Dec 11;13(24):2047. doi: 10.3390/cells13242047 (PMC11674128; doi:10.3390/cells13242047)
Supplement: Supplementary file 1 [file cells-13-02047-s001.zip › cells-3343439-supplementary.pdf]

cells

MDPI

Supplementary Materials

A Biorefinery Approach Integrating Lipid and EPS Augmentation Along with Cr (III) Mitigation by *Chlorella minutissima*

Sonia Choudhary [1,2], Mansi Tiwari [1], Krishna Mohan Poluri [1,2,*]

[1] Department of Biosciences and Bioengineering, Indian Institute of Technology Roorkee, Uttarakhand247667, India; schoudhary1@bt.iitr.ac.in (S.C.); mansi_t@bt.iitr.ac.in (M.T.)
[2] Centre for Transportation System, Indian Institute of Technology Roorkee, Uttarakhand 247667, India
* Correspondence: krishna.poluri@bt.iitr.ac.in[1]

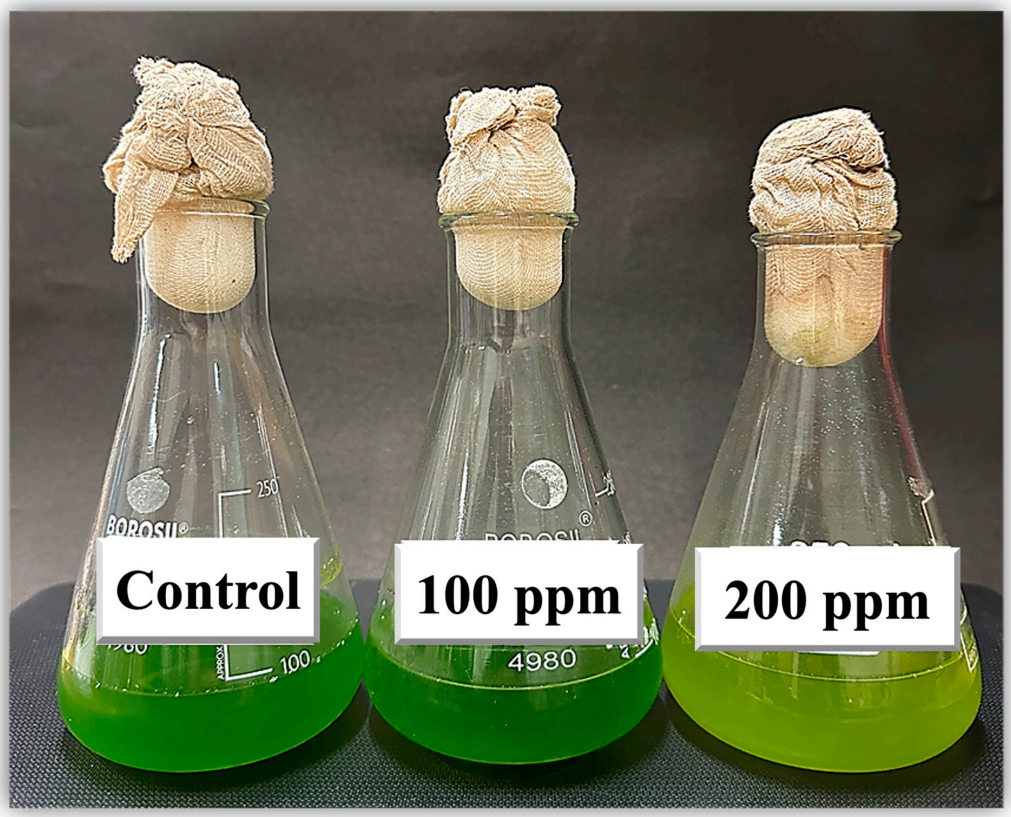

Supplementary Figure S1. Cultures of *Chlorella minutissima* cultivated in control (BBM) and at 100 and 200 ppm of Cr (III) spiked in BBM for 12 days.

Supplementary Table S1. Composition of Bold's basal media (BBM) and micronutrient solution.

Constituent Chemicals	Quantity (g/L)
Sodium nitrate ($NaNO_3$)	0.25
Potassium dihydrogen phosphate (KH_2PO_4)	0.175
Potassium hydrogen phosphate(K_2HPO_4)	0.075
Magnesium sulphate heptahydrate ($MgSO_4{\cdot}7H_2O$)	0.075
Sodium chloride (NaCl)	0.025
Calcium chloride dihydrate ($CaCl_2.2H_2O$)	0.025
Iron sulphate heptahydrate ($FeSO_4{\cdot}7H_2O$)	0.005
Disodium ethylenediaminetetraacetic acid, Na_2-(EDTA)	0.005
Micronutrient solution – 1 mL	

Supplementary Table S2. Composition of micronutrient solution.

Constituent Chemicals	Quantity (g/L)
Boric acid (H_3BO_3)	2.86
Zinc sulphate heptahydrate ($ZnSO_4{\cdot}7H_2O$)	0.222
Copper sulphate pentahydrate ($CuSO_4{\cdot}5H_2O$)	0.079
Manganese chloride tetrahydrate ($MnCl_2{\cdot}4H_2O$)	1.81
Cobalt nitrate hexahydrate (Co $(NO_3)_2{\cdot}6H_2O$)	0.041
Sodium molybdate dehydrate ($Na_2MoO_4.2H_2O$)	0.390

Supplementary Table S3. Empirical formulas for calculating biodiesel physical properties.

S. No.	Physical Property	Empirical Formula
1.	Saponification value (SV)	∑ 560 (% FA)/ Mw
2.	Iodine values (IVs)	∑ (254 db × % FA) / Mw
3.	Cetane number (CN)	46.3 + 5.458 / SV – (0.255 × IV)
4.	Degree of unsaturation (DU) (%)	% MUFA + (2 × % PUFA)
5.	Long chain saturation factor (LCSF)	(0.1 × C16) + (0.5 × C18)
6.	Cold filter plugging properties (CFPPs)	(3.417 × LCSF) – 16.477
7.	High heating value (HHV)	∑ % FA (46.19 – (1794/Mw) – 0.21 × db)
8.	Oxidative stability (h)	117.9295 / (% C18:2 + % C 18:3) + 2.5905

Where Mw = molecular mass of each fatty acid component, db = number of double bonds, FA = % of each fatty acid component, % MUFA = weight % of monounsaturated fatty acids, PUFA = weight % of polyunsaturated fatty acids.

Supplementary Table S4. Comparative analysis of IC_{50} values and Cr (III) removal efficiency (%) by different microalgae.

Microalgae Species	IC_{50} Value (ppm)	Removal (%)	Biomass yield (g/L)	Lipid Content (%)	References
C. minutissima	83.87 ppm	96.5	0.02492 g/L	30.29%	[1]
C. sorokiniana	189.21 ppm	97.1%	0.08932 g/L	28 %	[2]
Parachlorella kessleri R-3	0.5 ppm	72.2 %	1.04 g/L	48 %	[6]
Dictyosphaerium chlorelloides	14.27 ppm	-	-	-	[3]
Scenedesmus quadricauda	-	98.3 %	-	-	[4]
Chlorella sorokiniana	10 ppm	96 %	-	-	[5]
Chlorella minutissima	100 ppm	98 %	0.83196 g/L	32.65 %	Present study
	200 ppm	92 %	0.69966 g/L	42.67 %	Present study

Supplementary Table S5. A comprehensive examination of EPS biosynthesis and its carbohydrate content in the context of heavy metal exposure.

Microalgae species	Heavy metal	EPS content (mg/L)	Carbohydrate content (mg/L)	Reference
Parachlorella kessleri	-	12.49 mg/L	0.635 mg/L	[15]
Chlorella vulgaris	-	10.42 mg/L	0.577 mg/L	[15]
C. vulgaris	Cd (II)	17.5 mg/L	2.48 mg/L	[16]
Chlorella sp.	-	52 mg/L	23 mg/L	[17]
Scenedesmus sp. IITRIND2	Cd (II)	78mg/L	18 mg/L	[10]
Thalassiosira pseudonana	Hg (II)	7.6 mg/L	-	[18]
C. minutissima	Cr (III)	34.29 mg/L	16.78 mg/L	Present Study
		48.578 mg/L	19.58 mg/L	Present Study

Supplementary Table S6. Summary of lipid accumulation in response to different heavy metal stress in microalgal species.

Microalgae species	Heavy metal	Concentration (ppm)	Lipid content (%)	References
C. vulgaris	Cu	2.5 ppm	14.20 %	[7]
C. vulgaris	Cd	5 ppm	9.67 %	[7]
C. vulgaris	Zn	50 ppm	13.62 %	[7]
Scenedesmus quadricauda	Cr (VI)	5 ppm	13.2 %	[8]
Scenedesmus quadricauda	Cr (VI)	10 ppm	17.4 %	[8]
Monoraphidium sp. QLY-1	Cd	9 ppm	52.78 %	[9]

P. kessleri R-3	Cd (II)	0.5 ppm	59.9 %	[6]
Scenedesmus sp. IITRIND2	Cd (II)	31.7 ppm	33%	[10]
P. kessleri R-3	Cu (II)	0.5 ppm	65.1 %	[6]
Chlorella thermophilia	As (II)	15 ppm	-	[11]
P. kessleri R-3	Pb (II)	0.5 ppm	43.3 %	[6]
Chlorella vulgaris	Cu	2.5 ppm	14.20 %	[7]
Chlorella sp.	Cd (II)	7 ppm	-	[12]
C. pyrenoidosa	Fe	6.7 ppm	56.6 %	[13]
Chlorella sp.	Cr (VI)	0.5 ppm	54.17 %	[14]
Chlorella minutissima	Cr (III)	100 ppm	32.65 %	Present Study
		200 ppm	42.67 %	Present Study

References:

1. Chakravorty, M.; Jaiswal, K.K.; Bhatnagar, P.; Parveen, A.; Upadhyay, S.; Vlaskin, M.S.; Alajmi, M.F.; Chauhan, P.; Nanda, M.; Kumar, V. Exogenous GABA supplementation to facilitate Cr (III) tolerance and lipid biosynthesis in *Chlorella sorokiniana*. *J. Environ. Manag.* **2024**, *355*, 120441. https://doi.org/10.1016/j.jenvman.2024.120441.
2. Khoubestani, R.S.; Mirghaffari, N.; Farhadian, O. Removal of three and hexavalent chromium from aqueous solutions using a microalgae biomass-derived biosorbent. *Environ. Prog. Sustain. Energy* **2014**, *34*, 949–956. https://doi.org/10.1002/ep.12071.
3. Athira, K.; Sathish, A.; Nithya, K.; Guhananthan, A. Corn cob immobilised *Chlorella sorokiniana* for the sequestration of chromium ions from aqueous solution. *Mater. Today: Proc.* **2020**, *33*, 2148–2155. https://doi.org/10.1016/j.matpr.2020.03.151.
4. Song, X.; Kong, F.; Liu, B.-F.; Song, Q.; Ren, N.-Q.; Ren, H.-Y. Lipidomics analysis of microalgal lipid production and heavy metal adsorption under glycine betaine-mediated alleviation of low-temperature stress. J. Hazard. Mater. 2024, 480, 135831. https://doi.org/10.1016/j.jhazmat.2024.135831.
5. El-Naggar, A. H., Sheikh, H. M. Response of the green microalga *Chlorella vulgaris* to the oxidative stress caused by some heavy metals. *Life Sci. J.*, *11(10)*, 1349-1357. (**2014**). doi:10.7537/marslsj111014.197
6. Kafil, M.; Berninger, F.; Koutra, E.; Kornaros, M. Utilization of the microalga *Scenedesmus quadricauda* for hexavalent chromium bioremediation and biodiesel production. **2022**, *346*, 126665. https://doi.org/10.1016/j.biortech.2021.126665.
7. Ganguly, A.; Nag, S.; Bhowmick, T.K.; Gayen, K. Bioremediation of chromium(VI) and arsenic(III) using isolated microalgal (*Chlorella thermophilia*): Analysis of growth, biomolecular compositions (carbohydrate, protein, chlorophyll) and biosorption kinetics. *Algal Res.* **2024**, *82*. https://doi.org/10.1016/j.algal.2024.103635.
8. Duque-Granda, D.; Montoya-Vallejo, C.; Botero-Botero, L.R. Cadmium (Cd) tolerance evaluation of three strains of microalgae of the genus *Ankistrodesmus, Chlorella* and *Scenedesmus*. *Rev. Fac. De Ing. De Antioquia* **2019**, 88–95. https://doi.org/10.17533/udea.redin.20190523.
9. Nanda, M.; Jaiswal, K.K.; Kumar, V.; Vlaskin, M.S.; Gautam, P.; Bahuguna, V.; Chauhan, P. Micro-pollutant Pb(II) mitigation and lipid induction in oleaginous microalgae *Chlorella sorokiniana* UUIND6. *Environ. Technol. Innov.* **2021**, *23*. https://doi.org/10.1016/j.eti.2021.101613.
10. Liu, Y.; Zhan, J.-J.; Hong, Y. Effects of metal ions on the cultivation of an oleaginous microalga *Chlorella sp.*. *Environ. Sci. Pollut. Res.* **2017**, *24*, 26594–26604. https://doi.org/10.1007/s11356-017-0258-x.

11. Ciempiel, W.; Czemierska, M.; Szymańska-Chargot, M.; Zdunek, A.; Wiącek, D.; Jarosz-Wilkołazka, A.; Krzemińska, I. Soluble Extracellular Polymeric Substances Produced by *Parachlorella kessleri* and *Chlorella vulgaris*: Biochemical Characterization and Assessment of Their Cadmium and Lead Sorption Abilities. *Molecules* **2022**, *27*, 7153. https://doi.org/10.3390/molecules27217153.
12. Chen, B.; Li, F.; Liu, N.; Ge, F.; Xiao, H.; Yang, Y. Role of extracellular polymeric substances from *Chlorella vulgaris* in the removal of ammonium and orthophosphate under the stress of cadmium. *Bioresour. Technol.* **2015**, *190*, 299–306. https://doi.org/10.1016/j.biortech.2015.04.080.
13. Wang, M.; Kuo-Dahab, W.C.; Dolan, S.; Park, C. Kinetics of nutrient removal and expression of extracellular polymeric substances of the microalgae, *Chlorella sp*. and *Micractinium sp.*, in wastewater treatment. *Bioresour. Technol.* **2014**, *154*, 131–137. https://doi.org/10.1016/j.biortech.2013.12.047.
14. Zhang, D.; Lee, D.-J.; Pan, X. Desorption of Hg(II) and Sb(V) on extracellular polymeric substances: Effects of pH, EDTA, Ca(II) and temperature shocks. *Bioresour. Technol.* **2012**, *128*, 711–715. https://doi.org/10.1016/j.biortech.2012.10.089.
